# Transcriptome Analysis of Arbuscular Mycorrhizal *Casuarina glauca* in Damage Mitigation of Roots on NaCl Stress

**DOI:** 10.3390/microorganisms10010015

**Published:** 2021-12-23

**Authors:** Yihan Wang, Fengxin Dong, Ming Tang

**Affiliations:** 1 College of Forestry, Northwest A&F University, Xianyang 712100, China; 2018060256@nwafu.edu.cn (Y.W.); 2018060258@nwsuaf.edu.cn (F.D.); 2 Guangdong Laboratory for Lingnan Modern Agriculture, State Key Laboratory of Conservation and Utilization of Subtropical Agro-Bioresources, College of Forestry and Landscape Architecture, South China Agricultural University, Guangzhou 510642, China

**Keywords:** arbuscular mycorrhizal fungi, *Casuarina glauca*, transcriptome analysis, salt stress

## Abstract

*Casuarina glauca* grows in coastal areas suffering long-term damage due to high salt stress. Arbuscular mycorrhizal fungi (AMF) can colonize their roots to alleviate the effects of salt stress. However, the specific molecular mechanism still needs to be further explored. Our physiological and biochemical analysis showed that *Rhizophagus irregularis* inoculation played an important role in promoting plant growth, regulating ion balance, and changing the activity of antioxidant enzymes. Transcriptome analysis of roots revealed that 1827 differentially expressed genes (DEGs) were affected by both *R. irregularis* inoculation and NaCl stress. The enrichment of GO (Gene Ontology) and KEGG (Kyoto Encyclopedia of Genes and Genomes) showed that most of these DEGs were significantly enriched in ion transport, antioxidant enzyme activity, carbohydrate metabolism, and cell wall. *HAK5*, *KAT3*, *SKOR*, *PIP1-2*, *PER64*, *CPER*, *GLP10*, *MYB46*, *NAC43*, *WRKY1*, and *WRKY19* were speculated to play the important roles in the salt tolerance of *C. glauca* induced by *R. irregularis*. Our research systematically revealed the effect of *R. irregularis* on the gene expression of *C. glauca* roots under salt stress, laying a theoretical foundation for the future use of AMF to enhance plant tolerance to salt stress.

## 1. Introduction

Soil salinization is one of the main abiotic stresses that endanger plant growth [1]. At present, more than 20% of irrigated land in the world is facing the impact of soil salinization [2]. NaCl stress is mainly due to the accumulation of a large amount of Na^+^ and Cl^−^ in the rhizosphere of plants, which hinders the absorption of water and other mineral ions by the plant roots, thereby inhibiting the growth of plants [3,4,5]. It even causes dehydration and the death of plants at high concentrations [6,7]. To adapt to soil salinization and maintain growth, plants have evolved a series of strategies, including increasing antioxidant enzyme activity, removing excess reactive oxygen species (ROS) [8], selectively absorbing other ions to maintain the osmotic potential [6], and producing signal substances by changing the level of the hormone [9]. However, plants cannot fully cope the soil salinization only by themselves.

Roots tend to establish a symbiotic relationship with many microorganisms in the soil to deal with the negative effects of salt stress [10]. Arbuscular mycorrhizal fungi (AMF) are obligate symbiotic fungi that can establish a symbiotic relationship with more than 80% of the plants on the land [11]. By establishing a symbiotic relationship with AMF, plants absorb a larger range of water and minerals to meet their growth needs [12], providing organic matter for AMF to maintain the symbiotic relationship with them [13]. At present, studies have shown that AMF can alleviate the negative effects of salt stress to promote plant growth [12,14,15]. The increase in plant salt tolerance caused by AMF is attributed to the more effective absorption of water and nutrients [16], better osmotic state [17], protection of enzyme activity [11], increased photosynthesis [18], and alleviation of ion imbalance [19]. Exploring their molecular mechanism is an important step in understanding the salt tolerance induced by AMF. However, such research is limited.

*Casuarina glauca* can tolerate a high concentration of salt while maintaining its growth [20]. AMF can establish a symbiotic relationship to alleviate the negative effects of salt stress on *C. glauca* [21]. At present, studies have shown that inoculating *Casuarina* spp. seedlings with AMF can significantly increase the biomass [22], the ratio of other ions to Na^+^ [23], water absorption, and antioxidant enzyme activity [22,24]. However, the specific molecular mechanism is unclear. RNA sequencing (RNA–seq) is an effective method for studying the transcriptome, which is helpful to explore the molecular mechanism of stress tolerance [25,26]. This kind of research has been carried out in several salt-tolerant plant species, such as *Spartina alterniflora* [27], *Populus euphratica* [28], and *Beta vulgaris* [25].

Roots, colonized by AMF, are the first part to be stressed during salt stress. Hence, this article focused on the effects of NaCl stress and *Rhizophagus irregularis* on roots. We used RNA-seq to identify the transcriptome profile of *C. glauca* roots under different conditions, revealing the molecular mechanism of *R. irregularis* mediated NaCl tolerance. Through the transcriptome analysis, the research attempted to determine the key NaCl stress response pathways and genes mediated by *R. irregularis*. These results contributed to understanding the mechanism of salt tolerance induced by *R. irregularis*, laying the foundation for further improving the salt tolerance of plants.

## 2. Materials and Method

### 2.1. Plant Material

Seeds of *C. glauca* were provided by the Research Institute of Tropical Forestry, Chinese Academy of Forestry (Guangzhou, China). After surface sterilization in 75% ethanol solution for 30 s and 5% NaClO solution for 10 min, washing three times with sterile water, and being placed on a sterile filter paper to absorb the water, seeds were placed on the woody plant medium (WPM) to germinate into seedlings, then cultured for 2 months. A best-growing seedling was selected to prepare clonal plants. The leaves of that were divided into several 2 cm sections, and placed on WPM differentiation medium to differentiate into buds (containing 1.5 mg·L^−1^ 6-benzyl amino purine, 6-BA; 0.5 mg·L^−1^ indole acetic acid, IAA). The buds were cut off and inserted in WPM rooting medium (containing 1 mg·L^−1^ IAA) for rooting. Cultivation conditions: 16 h light daily, light intensity 3000 lux.

### 2.2. Experimental Design

The experiment was conducted in a completely randomized design with a factorial combination of 2 × 2 (salt stress, AMF inoculum) with 3 replicates. There were two levels of salt stress (0 and 600 mM NaCl, the screening criteria were shown in Figure S1) and two AMF treatments (NM: no AMF inoculum; AM: inoculation with *R. irregularis*). For each treatment, twelve plants were combined into one replicate.

### 2.3. Mycorrhizal Inoculum

The *R. irregularis* (BGC BJ09) was obtained from the Beijing Academy of Agriculture and Forestry Sciences (Beijing, China) and multiplied by *Lycopersicon esculentum* Mill. in sterilized sand. The inoculum contained spores (approximately 44 spores per gram), hyphae, and colonized root pieces. Each AM treatment was treated with 40 g inoculum. Each NM treatment received the same amount of autoclaved inoculum.

### 2.4. Growth Medium

The soil (<2 mm) used in this study was collected at 0–20 cm depth from soil at Northwest A&F University. Sand (<2 mm) was also collected and washed until there was no soil in it. The soil and sand were sterilized after being mixed at a volume ratio of 1:2. The sterilized soil/sand mixture had a pH of 7.6 (1:5, soil:water, *w*/*v*) and contained 34.9 mg·kg^−1^ available nitrogen, 15.8 mg·kg^−1^ available phosphorous, 165.7 mg·kg^−1^ available potassium, 17.5 g·kg^−1^ organic matter, 132.2 mg·kg^−1^ soluble sodium, and 37.2 mg·kg^−1^ chloride.

### 2.5. Growth Conditions

Pots were each filled with 1 kg of sterilized soil/sand mixture. Inoculation with *R. irregularis* was performed when the clonal plants were transplanted into the pots. After 8 weeks, colonization was identified. The maximum water capacity of the sand and soil mixture (1 kg) was 250 mL. The content of 600 mM NaCl was 8.766 g. During the NaCl stress, 8.766 g NaCl was applied 6 times (1.461 g was applied every three days). The plants were harvested after another 4 weeks. The water capacity was maintained at maximum water capacity by weight every day. Each pot was irrigated with 50 mL Hoagland solution (containing 1/10 phosphate) every week. The clonal plants were grown in a greenhouse under natural light at a temperature of 25–35 °C and a relative humidity of 50–75%.

### 2.6. Plant Harvest

Height was measured with a flexible rule, ground diameter was measured with a digital caliper (Hengliang, Shanghai, China). Roots were divided into four parts after determining the fresh weight: the first part was used to determine AMF colonization; the second part was used to measure water content; the third part was used to measure element concentrations. After being ground into a powder in the liquid nitrogen, the fourth part was used for the RNA extraction, antioxidant enzyme activity determination, and malondialdehyde (MDA) concentration.

### 2.7. Mycorrhizal Colonization

After being stained with trypan blue [29], roots were used to determine mycorrhizal colonization by the gridline intersection method [30].

### 2.8. Plant Biomass and Water Content

The weight during the harvest process was regarded as the fresh weight. Roots were dried at 65 °C until constant weight to determine the dry weight. The dry weight and fresh weight were used to calculate the water content.

### 2.9. Concentrations and Contents of Na^+^, K^+^, Ca^2+^, and Cl^−^

After dried, roots were ground into powder. Na^+^, K^+^, and Ca^2+^ were extracted by HNO_3_ and a microwave digestion instrument (Milestone ETHOS, Milan, Italy), then measured using an atomic absorption spectrophotometer (PerkinElmer, Waltham, MA, USA). Cl^−^ was extracted in boiling water [31] and measured by AgCl turbidimetry [32]. Element contents were calculated by the water content and fresh weight. Ten grams of air-dried soil (<1 mm) were placed in a 100 mL conical flask, to which 50 mL of water was added. After shaking for 3 min, the mixture was filtered with a Buchner funnel to obtain a 5:1 water:soil leaching solution [33]. The concentrations of Na^+^ and Cl^−^ in soil were measured using the same methods used with roots.

### 2.10. Antioxidant Enzyme Activities

The method of superoxide dismutase (SOD) activity determination referred to the method of Mallick and Mohn [34]. Weigh 0.1 g of fresh root powder, add 4 mL of pH 7.8 pre-cooled phosphate buffer (PB), mix upside down, centrifuge at 4000 rpm, 4 °C for 10 min, transfer the supernatant to a new centrifuge tube. The reaction mixture was mixed with enzyme solution, 50 mM PB, 100 μM EDTA-Na_2_, 130 mM methionine (Met), 750 μM nitro blue tetrazolium (NBT), 20 μM riboflavin. The absorbance value was measured at 560 nm (A_560_). Take the amount of enzyme required to inhibit 50% of the NBT photoreduction reaction as an enzyme activity unit (U). SOD activity was calculated using the following formulas:

$$\mathrm{SOD}\;\mathrm{activity}\;(U\cdot g^{-1}\;\mathrm{FW}\cdot \min^{-1})=\frac{{(A}_{0}{\;-\;A}_{s}{)\;\times \;V}_{t}}{A_{0}\;\times \;0{.5\;\times \;\mathrm{FW}\;\times \;V}_{s\;}\times \;t}$$

where *A*_0_ is the absorbance of control tube under light; *A_s_*, absorbance of sample tube; *V_t_*, total volume of sample extract (mL); *V_s_*, volume of enzyme solution during measurement (mL); *t*, light time of color reaction (min); FW, root fresh weight (g).

The method of peroxidase (POD) activity determination referred to the method of Fang and Kao [35]. Fresh root powder (0.1 g) was mixed with 4 mL pre-cooled distilled water. After being centrifuged at 4000 rpm, 4 °C for 10 min, the supernatant was the enzyme solution. The reaction mixture was mixed with enzyme solution, 0.1% guaiacol, and 0.18% H_2_O_2_. The total volume was 6.3 mL. The absorbance value was measured at 470 nm (*A*_470_). POD activity was calculated using the following formulas:

$$\mathrm{POD}\;\mathrm{activity}\;(\mu g\cdot g^{-1}\;\mathrm{FW}\cdot \min^{-1})=\frac{{(X\;-\;X}_{0}{)\;\times \;V}_{t}}{{\mathrm{FW}\;\times \;V}_{s\;}\times \;t}$$

where *X*_0_ denotes the content of 4-Methoxyphenol in the control tube (μg); *X,* 4-Methoxyphenol content in the sample tube calculated from the standard curve (μg); *V_t_,* total volume of sample extract (mL); *V_s_,* volume of enzyme solution during measurement (mL); *t,* reaction time (min); FW, root fresh weight (g).

### 2.11. MDA Concentration

MDA concentration was determined as described by Kramer et al. [36]. Weigh 0.1 g of fresh root powder, add 4 mL 10% trichloroacetic acid (TCA), mix upside down, centrifuge at 4000 rpm, 4 °C for 10 min, transfer the supernatant to a new centrifuge tube. After adding 0.6% thiobarbituric acid (TBA), the mixture was heated in boiling water for 30 min. The absorbance value was measured at 450, 532, and 600 nm (*A*_450_, *A*_532_, *A*_600_). MDA concentration was calculated using the following formulas:

$$\mathrm{MDA}\;\mathrm{concentration}\;(\mathrm{mmol}\cdot g^{-1}\;\mathrm{FW})=[6.452\;\times \;\left(A_{532}{\;-\;A}_{600}\right)\;-\;0{.559\;\times \;A}_{450}]\;\times \;\frac{V_{t}}{V_{s\;}\times \;\mathrm{FW}}$$

where *V_t_* denotes the total volume of sample extract (mL); *V_s_,* volume of enzyme solution during measurement (mL); FW, root fresh weight (g).

### 2.12. RNA Extraction, RNA–seq, and Bioinformatics Analysis

Total RNA was extracted from roots using the E.Z.N.A Plant RNA Kit R6827-01 (Omega Bio-Tek, Norcross, GA, USA). The RNA samples were accepted when 260/280 ratios were determined as 1.9–2.1 using a Nano Photometer^®^ spectrophotometer (IMPLEN, Westlake Village, CA, USA), with RIN value (RNA integrity number) >6.0 obtained using RNA Nano 6000 Assay Kit of the Bioanalyzer 2100 system (Agilent Technologies, CA, USA). Then, the RNA was sent to the Shanghai Personal Biotechnology Cp. Ltd. for RNA–Seq analysis (Personalbio, Shanghai, China). FastQC (version 0.11.8) (https://www.bioinformatics.babraham.ac.uk/projects/fastqc/, accessed on 8 January 2021) and Cutadapt (version 1.16) [37] were used to control the quality of original data. Trinity software (version 2.5.1) [38] was used to splice the transcript according to the default parameters. The longest sequence was used as the reference transcript sequence (Unigene) for subsequent analysis. The obtained Unigene was input in NR (NCBI non-redundant protein sequences), GO (Gene Ontology) [39], KEGG (Kyoto Encyclopedia of Genes and Genomes) [40], eggNOG (evolutionary genealogy of genes: Non-supervised Orthologous Groups) [41], Swiss-Prot (https://www.ebi.ac.uk/uniprot/, accessed on 8 January 2021), and Pfam (http://pfam.xfam.org/, accessed on 8 January 2021) databases to compare the corresponding function annotations. RSEM software (version 1.2.15) [42] was used to perform quantitative analysis on Unigene. The obtained data were calculated using DEGseq (version 1.32.0) [43] in R language according to |log2foldchang| > 1 and *p*-value < 0.05 to obtain differentially expressed genes (DEGs). Expression levels were estimated by fragments per kilobase of exon model per million mapped fragments (FPKM). The topGO (version 2.32.0) [44] software was used for GO enrichment analysis according to default parameters, while KEGG enrichment analysis was analyzed using the Parsons online analysis website (https://www.genescloud.cn/chart/KEGGenrich, accessed on 8 January 2021) according to default parameters.

### 2.13. Gene Expression Based on Quantitative Real-Time PCR (qRT–PCR)

RNA was reverse transcribed to cDNA by a TIANScript RT Kit (TIANGEN Bio, Beijing, China). Eight genes, i.e., TRINITY_DN2270_c0_g2 (High-affinity K^+^ transporter, *HAK5*), TRINITY_DN715_c1_g1 (K^+^ affinity transporter, *KAT3*), TRINITY_DN7186_c0_g3 (Shaker-type K^+^ outward rectifier, *SKOR*), TRINITY_DN5565_c0_g1 (Na^+^/Ca^2+^ exchanger-like protein, *NCL*), TRINITY_DN3109_c3_g1 (Peroxidase 64, *PER64*), TRINITY_DN31653_c0_g1 (Putative cytochrome c peroxidase, *CPER*), TRINITY_DN13561_c0_g1 (Germin-like protein, *GLP10*), and TRINITY_DN22047_c0_g4 (Polyubiquitin, *TU20*), related to ion transport and antioxidant enzymes were randomly selected. The gene sequences were obtained from the assembly of the *C. glauca* RNA–seq using Illumina HiSeq technology (PRJNA690646). The primers used in the qRT–PCR, as described in Table S1, were designed with the NCBI Primer-BLAST tool. qRT–PCR was conducted using a CF96X real-time PCR system (Bio-Rad, Hercules, CA, USA). Each reaction mixture was 10 µL and contained 1 µL diluted cDNA template, 0.5 µL forward and reverse primers (10 μM), 5 µL ChamQ SYBR qPCR Master Mix (Vazyme, Nanjing, China), and 3 µL sterilized ddH_2_O. The two-step qRT–PCR was run as follows: 30 s denaturation at 95 °C, 40 cycles of denaturation at 95 °C for 10 s, annealing at the annealing temperature for 10 s, extension at 72 °C for 20 s, followed by heating from 65 to 95 °C at a rate of 0.5 °C every 5 s. All samples were performed two technical replicates. The relative expression level was determined using the $2^{-\Delta \Delta C_{T}}$ method [45].

### 2.14. Statistical Analyses

SPSS 26 statistical software (SPSS Inc., Chicago, IL, USA) was used for statistical analyses. All data were analysed by one-way ANOVA, post hoc comparisons (Tukey’s test, *p* < 0.05, *n* = 3), and two-way ANOVA (NaCl stress, inoculation of *R. irregularis*, and their interaction). Z-score was used to standardize the data and make a correlation curve for qRT–PCR verification and RNA-Seq results. Figures were constructed with Origin 2020 (Origin Lab, Northampton, MA, USA). Heatmaps were generated with MetaboAnalyst (https://www.metaboanalyst.ca/?tdsour%cetag=s_pctim_aiomsg, accessed on 3 July 2021).

## 3. Results

### 3.1. Physiological and Biochemical Analysis in C. glauca Roots

Under NaCl stress, inoculation with *R. irregularis* increased the fresh weight of shoots by 369%, the fresh weight of roots by 265%, the height by 116%, and the ground diameter by 77%. In mycorrhizal roots, NaCl stress reduced the colonization of arbuscule from 39% to 13% and increased the colonization of vesicle and spore from 34% to 57%, but the difference in colonization of hypha was not significant (*p* = 0.053988). Under NaCl stress, inoculation with *R. irregularis* increased the Na^+^, Cl^−^ content of roots by 234% and 260%, but decreased the Na^+^, Cl^−^ content of soil by 37% and 18%. The Na^+^, Cl^−^ concentrations of roots were increased by NaCl stress. However, inoculation with *R. irregularis* decreased the Na^+^, Cl^−^ concentration of roots by 12% and 5% under NaCl stress. The contents of K^+^ and Ca^2+^ in roots were reduced by NaCl stress. However, under NaCl stress, inoculation with *R. irregularis* increased the content of K^+^ and Ca^2+^ in roots by 338% and 183% and decreased Na^+^/K^+^ and Na^+^/Ca^2+^ of roots by 24% and 31%. Under NaCl stress, inoculation with *R. irregularis* decreased the MDA concentration of roots by 24%. NaCl stress increased the activity of POD and SOD. However, under NaCl stress, inoculation with *R. irregularis* increased the activity of SOD in roots by 63%, but the activity of POD was almost the same (*p* = 0.999971) (Table 1).

### 3.2. Summary of Sequencing Results

In this study, RNA–seq was performed on 12 samples, and an average of 45,133,745 raw reads was obtained. The average fuzzy base (N) was 0.000348%, the average Q20 was 97.67%, and the average Q30 was 93.79%. After strictly removing low-quality sequences, an average of 41,811,754 clean reads was obtained (Table S2).

After using Trinity software to splice and remove redundant sequences, a total of 58,988 sequences were obtained. The maximum sequence length was 20,358 bp, the average length was 1431.28 bp, the N50 value was 2832 bp, the N90 value was 525 bp, and the GC content was 40.77% (Table S3).

The obtained Unigene was annotated with gene function. There were 31,457, 15,702, 14,075, 19,496, 30,628, and 26,164 sequences respectively annotated to NR, GO, KEGG, eggNOG, Pfam, and the Swiss-Prot databases (Table S4).

### 3.3. Analysis of DEGs

**Figure 1:**
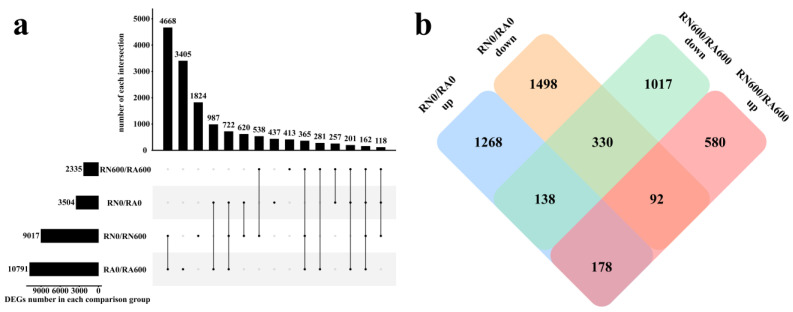
Upset diagram (**a**) and Venn diagram (**b**) of DEGs. R, roots; N, nonmycorrhizal; A, inoculated with *R. irregularis*; 0, no NaCl stress; 600, 600 mM NaCl stress. In Figure a, a point on the abscissa represents the number of unique DEGs annotated by the database; vertically connected points indicate the number of common DEGs annotated by multiple databases.

Under NaCl stress free condition, there were 3504 DEGs in RN0/RA0. Under non-inoculated condition, RN0/RN600 had a total of 9017 DEGs, indicating that the number of *C. glauca* genes effected by NaCl stress was significantly greater than the number of genes effected by *R. irregularis*. There were 10,791 DEGs in RA0/RA600, which was more than non-mycorrhizal *C. glauca*, indicating that mycorrhizal *C. glauca* regulated more genes to help roots alleviate the damage of NaCl stress, or severe growth inhibition or transcriptional regulation has been damaged by NaCl stress in non-mycorrhizal *C. glauca* (Figure 1a). Under the conditions of inoculation and NaCl stress, RN600/RA600 had 850 DEGs (580, 92, and 178) up-regulated and 1485 DEGs (1017, 330, and 138) down-regulated. These genes induced by *R. irregularis* under NaCl stress may help *C. glauca* to alleviate the damage of NaCl stress (Figure 1b).

A total of 14,998 (4668, 3405, 1824, 987, 722, 620, 538, 437, 413, 365, 281, 257, 201, 162, and 118) DEGs were identified in the four comparison groups. In the comparison group with and without *R. irregularis* (RN0/RN600, RA0/RA600), NaCl stress adjusted a total of 13,891 DEGs (4668, 3405, 1824, 987, 722, 620, 538, 365, 281, 201, 162, and 118), of which a total of 5917 DEGs (4668, 722, 365, and 162) were found in both RN0/RN600 and RA0/RA600. The number of common DEGs accounted for about half of the total number of DEGs in each comparison group, showing the variable mechanism of *C. glauca* to NaCl stress during *R. irregularis* inoculation (Figure 1a).

Most of the DEGs under NaCl stress were different from those without NaCl stress. Hence, 508 DEGs (330 and 178) were not affected by NaCl stress to maintain the original difference trend, and these genes may be the key genes to maintain the symbiotic relationship. Moreover, 230 DEGs (138 and 92) showed opposite trends with and without NaCl stress, and they may be involved in the regulation of symbiotic relationships and response to NaCl stress. In addition, 1597 new DEGs (1017 and 580) were induced by *R. irregularis* under NaCl stress, which may be the genes in mycorrhizal *C. glauca* that specifically respond to NaCl stress. Therefore, 1827 DEGs (1597 new DEGs and 230 DEGs with opposite trends) were speculated to be involved in the process of *C. glauca* in response to NaCl stress under the inoculation of *R. irregularis* (Figure 1b).

### 3.4. Analysis of GO, KEGG Enrichment

**Figure 2:**
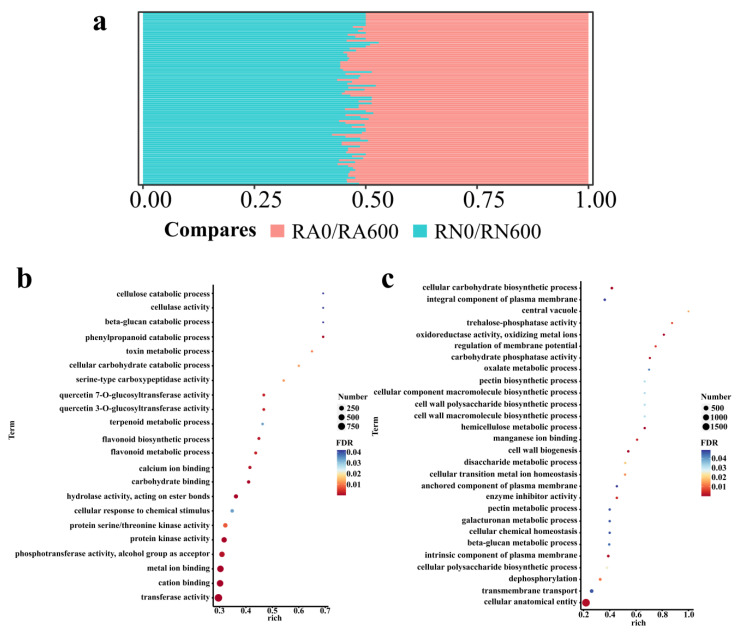
DEGs GO enrichment results in RA0/RA600 (**b**) and RN0/RN600 (**c**). R, roots; N, nonmycorrhizal; A, inoculated with *R. irregularis*; 0, no NaCl stress; 600, 600 mM NaCl stress. (**a**) The percentage of the DEGs number in each of the same 88 GO pathways in RA0/RA600 and RN0/RN600, compares represents the comparison group, the exact number and names of 88 GO pathways in RA0/RA600 and RN0/RN600 please refer to Table S5; (**b**) The bubble chart of 22 additional GO terms that were significantly enriched in RA0/RA600; (**c**) The bubble chart of 31 additional GO terms that were significantly enriched in RN0/RN600. Among them, the term represents the name of each channel, FDR represents the probability of enrichment, the number represents the number of DEGs contained in each channel, and rich represents the enrichment factor.

By enriching and comparing DEGs in RN0/RN600 and RA0/RA600 respectively, it’s helpful to clarify the similarities and differences between mycorrhizal and non-mycorrhizal *C. glauca* in response to NaCl stress. RN0/RN600 and RA0/RA600 were significantly enriched to 110 and 119 GO terms, respectively, indicating that no matter with or without *R. irregularis*, NaCl stress would have a wide range of effects on *C. glauca*. The number of GO terms that were significantly enriched after inoculation with *R. irregularis* was less than that of non-*R. irregularis*, indicating that mycorrhizal *C. glauca* was under less stress than non-mycorrhizal *C. glauca*, or genes upregulated by mycorrhizal *C. glauca* were well worked to enhance salt tolerance. Among them, 88 GO terms appear in RN0/RN600 and RA0/RA600, mainly related to redox (such as “oxidoreductase activity, acting on diphenols and related substances as donors, oxygen as acceptor”, “oxidoreductase activity”) and anti-oxidation (such as “hydrogen peroxide metabolic process”, “response to oxidative stress”), cell wall activity (such as “plant-type cell wall organization”, “cell wall macromolecule metabolic process”), and metabolic process (such as “secondary metabolite biosynthetic process”, “cellular carbohydrate metabolic process”), indicating that these pathways were the main pathways for *C. glauca* to respond to NaCl stress. In these major pathways, the number of DEGs enriched by RA0/RA600 was often greater than that of RN0/RN600, indicating that *R. irregularis* could regulate more genes to help *C. glauca* relieve the damage of NaCl stress (Figure 2a). In addition, there were unique GO terms which were additionally significantly enriched in these two comparison groups, such as carbohydrate metabolism (such as “cellular carbohydrate biosynthetic process”, “carbohydrate phosphatase activity”) and plasma membrane (such as “integral component of plasma membrane”, “anchored component of plasma membrane”) in RN0/RN600 and “metal ion binding”, “flavonoid biosynthetic process”, and “carbohydrate binding” in RA0/RA600 (Figure 2b,c). This showed that the methods used to alleviate the damage of NaCl stress between mycorrhizal and non-mycorrhizal *C. glauca* were different.

**Figure 3:**
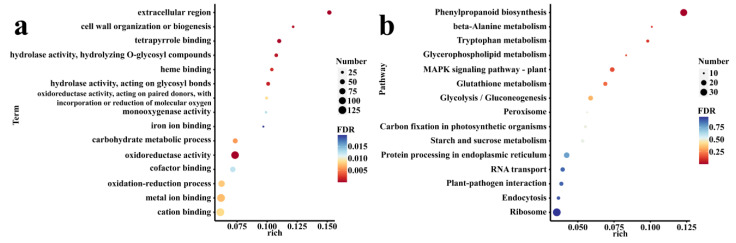
GO (**a**) and KEGG (**b**) enrichment of 1827 DEGs in response to NaCl stress induced by *R. irregularis*. (**a**) The bubble chart of the 15 GO terms enriched with the most DEGs; (**b**) the bubble chart of the 15 KEGG pathways enriched with the most DEGs. Among them, the term represents the name of each channel, FDR represents the probability of enrichment, the number represents the number of DEGs contained in each channel, and rich represents the enrichment factor.

To explore the role of *R. irregularis* in alleviating the damage of NaCl stress, 1827 DEGs induced by *R. irregularis* in the RN600/RA600 were subjected to GO and KEGG enrichment analysis (Figure 3). These DEGs were significantly enriched in 59 GO terms. Among them, the number of DEGs in metal ion binding and cation binding GO terms were the greatest. More DEGs were also significantly enriched in GO terms related to secondary metabolites (such as “secondary metabolite biosynthetic process”, “secondary metabolic process”), antioxidant (such as “hydrogen peroxide metabolic process”, “ROS metabolic process”), cell wall activities (such as “plant-type cell wall organization”, “cell wall macromolecule metabolic process”), and ion transport (such as “calcium ion transmembrane transporter activity”, “organic anion transmembrane transport”). Figure 3a presents the bubble chart of the 15 GO terms enriched with the most DEGs, implying the vigorous life activities in the mycorrhizal *C. glauca*.

In the KEGG enrichment analysis, all DEGs were enriched in 94 KEGG pathways. Among them, the most enriched DEGs in the “Ribosome” pathway also showed that the metabolism related to life activities in mycorrhizal *C. glauca* was relatively strong. The second enriched DEGs pathway is “phenylpropanoid biosynthesis”. In addition, more DEGs were also enriched in “MAPK signaling pathway-plant” and “plant-pathogen interaction”, implying that *C. glauca* is also actively maintaining an actively symbiotic relationship during NaCl stress. Figure 3b presents the bubble chart of the 15 KEGG pathways enriched with the most DEGs.

### 3.5. Response of DEGs Induced by R. irregularis under NaCl Stress

**Figure 4:**
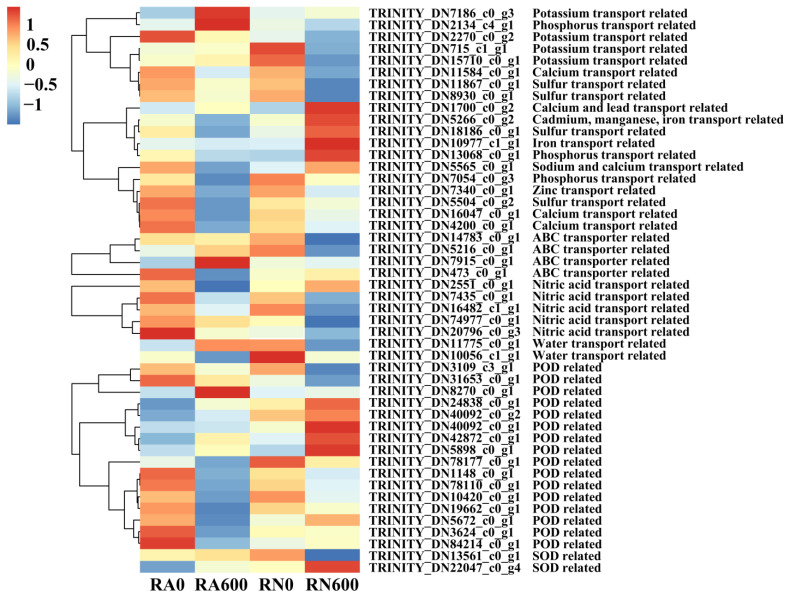
Heat map of DEGs related to ion transport and antioxidant enzymes in different treatments. R, roots; N, nonmycorrhizal; A, inoculated with *R. irregularis*; 0, no NaCl stress; 600, 600 mM NaCl stress.

To understand the molecular mechanism of *R. irregularis* in mitigating the damage of NaCl stress, the expression patterns related to ion balance transport and antioxidant enzymes were analyzed in RN600/RA600 (Figure 4). In RN600/RA600, only TRINITY_DN5565_c0_g1 (*NCL*) was found to be related to “sodium ion transmembrane transporter activity” and down-regulated, indicating that *R. irregularis* may inhibit the transmembrane transport of Na^+^. Three DEGs related to K^+^ transport were identified, namely TRINITY_DN2270_c0_g2 (*HAK5*), TRINITY_DN715_c1_g1 (*KAT3*), and TRINITY_DN7186_c0_g3 (*SKOR*). All of them were up-regulated by *R. irregularis* under NaCl stress. This implied that *R. irregularis* induced *C. glauca* to absorb a large amount of K^+^ to balance the Na^+^/K^+^ under NaCl stress. In addition, a small amount of DEGs were also enriched in transporter related to calcium, iron, manganese, zinc, etc., but most of them showed a down-regulated state. In terms of anion transport, DEGs were mainly enriched in sulfate (four DEGs) and phosphate (three DEGs) transport. These anions may be important elements to maintain the balance of anion and cation in *C. glauca* under NaCl stress.

In the POD-related pathways, 16 DEGs were enriched. However, only three of them were up-regulated, while all the rest were down-regulated, implying that the regulation of POD synthesis was different in mycorrhizal and non-mycorrhizal *C. glauca*. The expression of TRINITY_DN3109_c3_g1 (*PER64*) and TRINITY_DN31653_c0_g1 (*CPER*) was greatly increased by *R. irregularis* under NaCl stress, suggesting that *R. irregularis* may increase POD content by inducing this gene. In the pathway related to SOD synthesis, two DEGs were enriched and TRINITY_DN13561_c0_g1 (*GLP10*) was significantly up-regulated, which may be related to the substantial increase of SOD content in roots.

TRINITY_DN74977_c0_g1 (*NRT2-4*), TRINITY_DN7435_c0_g1 (*NPF4-6*), and TRINITY_DN16482_c1_g1 (*NPF1-2*) were nitrate transporter (NRT) related genes in *C. glauca* and up-regulated by *R. irregularis* under NaCl stress, indicating that *R. irregularis* induced them to promote N uptake and ion balance in *C. glauca*. At the same time, the expression of TRINITY_DN11775_c0_g1 (*PIP1-2*) and TRINITY_DN10056_c1_g1 (*PIP2-7*) was identified in RN600/RA600. Among them, TRINITY_DN11775_c0_g1 (*PIP1-2*) was significantly up-regulated by *R. irregularis*, suggesting that *R. irregularis* also played an important role in water transport under NaCl stress.

### 3.6. Exploration of Transcription Factor Family Induced by R. irregularis

**Figure 5:**
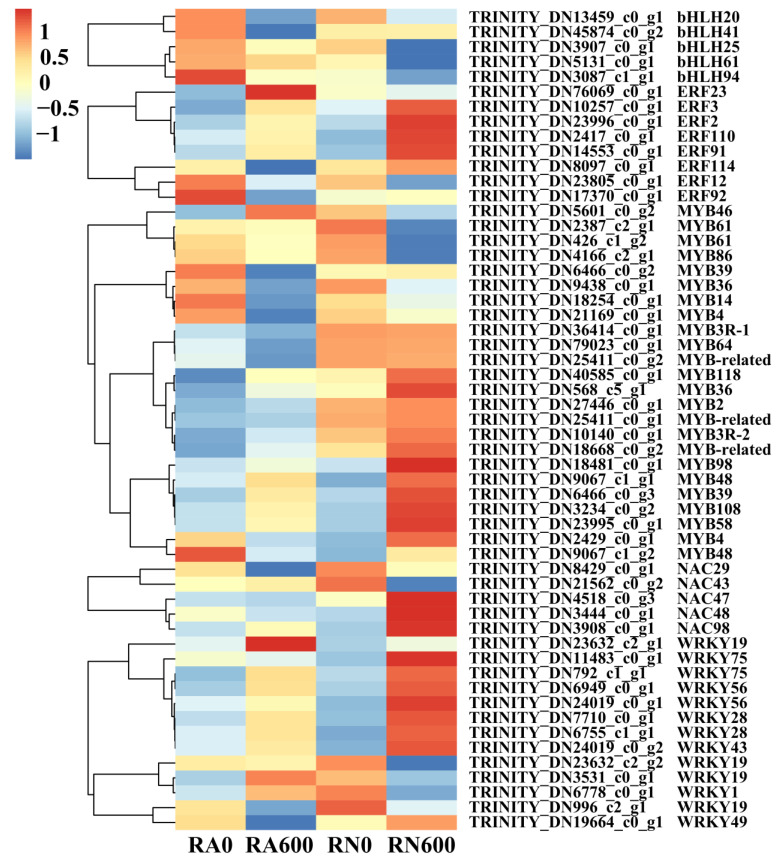
Heat map of DEGs related to transcription factors in different treatments. R, roots; N, nonmycorrhizal; A, inoculated with *R. irregularis*; 0, no NaCl stress; 600, 600 mM NaCl stress.

In RN600/RA600, 55 DEGs in bHLH, ERF, MYB, MYB related, NAC, and WRKY transcription factor families have been identified (Figure 5). In these families, most DEGs were down-regulated, maybe because the roots of mycorrhizal *C. glauca* were less affected by NaCl stress. The expressions of TRINITY_DN76069_c0_g1 (*ERF23*), TRINITY_DN5601_c0_g2 (*MYB46*), TRINITY_DN21562_c0_g2 (*NAC43*), TRINITY_DN6778_c0_g1 (*WRKY1*), and TRINITY_DN3531_c0_g1 (*WRKY19*) in mycorrhizal *C. glauca* were higher than that of non-mycorrhizal *C. glauca* under NaCl stress. This showed that these DEGs are important in NaCl tolerance of *C. glauca* induced by *R. irregularis*.

### 3.7. The qRT–PCR Verification

**Figure 6:**
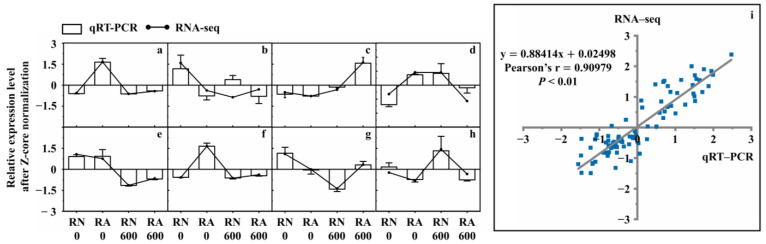
Correlation between qRT–PCR and RNA–Seq. R, roots; N, nonmycorrhizal; A, inoculated with *R. irregularis*; 0, no NaCl stress; 600, 600 mM NaCl stress. The relative expression level of each treatment was expressed as the change fold relative to RN0. (**a**) TRINITY_DN2270_c0_g2 (*HAK5*); (**b**) TRINITY_DN715_c1_g1 (*KAT3*); (**c**) TRINITY_DN7186_c0_g3 (*SKOR*); (**d**) TRINITY_DN5565_c0_g1 (*NCL*); (**e**) TRINITY_DN3109_c3_g1 (*PER64*); (**f**) TRINITY_DN31653_c0_g1 (*CPER*); (**g**) TRINITY_DN13561_c0_g1 (*GLP10*); (**h**) TRINITY_DN22047_c0_g4 (*TU20*). (**i**) Correlation curve of relative expression of 8 selected DEGs in roots between RNA–Seq and qRT–PCR. Standard error is represented by the error bars.

To validate RNA-Seq data, TRINITY_DN2270_c0_g2 (*HAK5*), TRINITY_DN715_c1_g1 (*KAT3*), TRINITY_DN7186_c0_g3 (*SKOR*), TRINITY_DN5565_c0_g1 (*NCL*), TRINITY_DN3109_c3_g1 (*PER64*), TRINITY_DN31653_c0_g1 (*CPER*), TRINITY_DN13561_c0_g1 (*GLP10*), and TRINITY_DN22047_c0_g4 (*TU20*) related to ion transport and antioxidant enzymes were randomly selected for qRT–PCR analysis. qRT-PCR and RNA-seq results of these eight genes were highly consistent (Pearson’s r = 0.90979, *p* < 0.01) (Figure 6). This indicated that the results of transcriptome analysis were highly reliable.

## 4. Discussion

Whether in crops (such as cucumber, corn, and wheat) [12,46,47] or forests (such as poplar, locust, and mulberry) [48,49,50], it has been confirmed that AMF can induce the host’s systemic tolerance to NaCl stress by the maintenance of ion balance and increase of biomass and antioxidase activity. As a typical saline-alkali plant, *C. glauca* can tolerate high-salt environments [51]. Symbiosis between AMF and roots plays an important role in salt tolerance of *C. glauca* [4]. Studies on *C. glauca* have shown that the salt tolerance induced by AMF may be attributed to an improvement in ion levels, photosynthesis, water absorption, and plant morphology [21]. This study further revealed the contribution of *R. irregularis* in NaCl tolerance of mycorrhizal *C. glauca* by physiological, biochemical, and transcriptome analysis under NaCl stress.

In this study, inoculation with *R. irregularis* could significantly increase biomass, ameliorate the ratio of Na^+^ to other metal cations, and increase antioxidant enzyme activity in *C. glauca* under NaCl stress. These findings are similar to those of Djighaly et al. [21], further confirming the importance of AMF in improving the salt tolerance of *C. glauca*. Under NaCl stress, the mycorrhizal *C. glauca* had a lower concentration but higher content of Na^+^ than non-mycorrhizal *C. glauca*, which caused the lower Na^+^ concentration in soil than non-mycorrhizal *C. glauca* but reduced the NaCl damage of mycorrhizal *C. glauca*. The results of transcriptome analysis showed that both NaCl stress and *R. irregularis* can induce a large number of genetic changes in *C. glauca*, and a total of 14,998 DEGs were identified. Compared with control, *R. irregularis* inoculation could cause changes in 3504 DEGs. Most of these DEGs were related to ion transport, hormones, and antioxidase activities. These data confirmed the construction of the molecular-level interaction relationship between *R. irregularis* and *C. glauca*. In addition, 1827 DEGs were specifically affected by *R. irregularis* inoculation. Most of these DEGs were significantly enriched in ion transport, antioxidase activity, carbohydrate metabolism, cell wall, and other processes, indicating that they were important in enhancing the NaCl tolerance of *C. glauca* induced by *R. irregularis*.

### 4.1. R. irregularis Inoculation Enhances the Ability to Remove the ROS of C. glauca under NaCl Stress

Plants can produce excessive ROS when they are exposed to NaCl stress [52]. If ROS cannot be removed in time, it will cause membrane lipid peroxidation and other harms, which will damage normal cells [53]. Therefore, plants have evolved a set of antioxidant enzyme protection systems (such as SOD and POD) and non-enzymatic protection systems (such as ascorbic acid and carotenoids) [46]. They can effectively remove ROS to ensure the normal development of plants [54]. This study explored the effect of *R. irregularis* inoculation on MDA concentration and activities of SOD and POD. MDA is a by-product of membrane lipid peroxidation, and its content is often proportional to ROS content [55]. The results of our research are similar to most of the mycorrhizal plants under NaCl stress [56,57]. The inoculation with *R. irregularis* could reduce the MDA concentration of *C. glauca* under NaCl stress, indicating that *R. irregularis* inoculation can reduce the ROS in *C. glauca* under NaCl stress. NaCl stress increased the activity of SOD and POD in roots. When *R. irregularis* inoculation was present under NaCl stress, it significantly increased the activity of SOD in roots, but the POD activity was almost the same, indicating that mycorrhizal *C. glauca* seems to prefer SOD removal in the way of removing ROS under NaCl stress. This was probably because the POD activity per unit of fresh weight has reached the maximum. However, because the biomass of mycorrhizal *C. glauca* was more than non-mycorrhizal *C. glauca*, the content of SOD and POD in mycorrhizal *C. glauca* was higher. From a molecular perspective, under NaCl stress, there were also differences in the antioxidant enzyme synthesis between mycorrhizal and non-mycorrhizal *C. glauca*. Among the 1827 DEGs induced by *R. irregularis* in the roots, three of the 16 DEGs related to POD synthesis were identified, and one of the two DEGs related to SOD synthesis was up-regulated. The expression of TRINITY_DN3109_c3_g1 (*PER64*), TRINITY_DN31653_c0_g1 (*CPER*), and TRINITY_DN13561_c0_g1 (*GLP10*) was greatly increased by *R. irregularis* inoculation under NaCl stress, indicating that these genes may be important in increasing the content of POD and SOD.

### 4.2. R. irregularis Inoculation Promotes the Ion Absorption Capacity of C. glauca under NaCl Stress

The root is the main organ for plants to absorb water and nutrients and it is also the first part damaged by salt [12]. Due to the presence of excess Na^+^ around the roots, it hinders the absorption of other essential elements by roots, effecting the ion homeostasis and inhibiting growth of plants [58]. As the important symbiotic fungi in roots, AMF are mainly used to replace root hairs and assist plants in selectively absorbing mineral nutrients [59]. In our research, we found that inoculation with *R. irregularis* could increase the contents of K^+^ and Ca^2+^ in *C. glauca*, reduce the Na^+^/K^+^ and Na^+^/Ca^2+^, and maintain the ion balance in *C. glauca*. From a molecular perspective, genes related to the transport of K^+^, Ca^2+^, SO_4_^2-^, and PO_4_^3-^ were induced by *R. irregularis* inoculation in mycorrhizal *C. glauca* under NaCl stress. These genes may be closely related to *R. irregularis* inoculation in maintaining ion balance of *C. glauca* under NaCl stress. Excessive Na^+^ can compete with K^+^ for binding sites on the lipid membrane around roots under NaCl stress, which hindered the absorption of K^+^ [60]. Potassium deficiency can not only disrupt the ion balance but also inhibit growth of plants [61]. Research by Estrada et al. [10] found that *R. irregularis* could increase the K^+^ content and reduce the Na^+^/K^+^ of corn under NaCl stress, which also indicated that this phenomenon was related to changes in *SKOR* expression. *HAK5* and *KAT3* can also be up-regulated by NaCl stress to promote the absorption of K^+^ by plants [62,63]. In our study, it was found that *R. irregularis* inoculation significantly increased TRINITY_DN2270_c0_g2 (*HAK5*), TRINITY_DN715_c1_g1 (*KAT3*), and TRINITY_DN7186_c0_g3 (*SKOR*) expression under NaCl stress. They may be the key genes for mycorrhizal *C. glauca* to maintain the Na^+^/K^+^ balance, and it also indicates that mycorrhizal *C. glauca* can induce the *HAK5* and *KAT3* to promote the absorption of K^+^ in *C. glauca* under NaCl stress.

Plant NRT participates in growth and the absorption and operation of nitrate nitrogen and other mineral ions of roots by regulating hormone transport and signal transduction, or as other ion transporters directly [64]. It also effects the plant tolerance under ion stresses [65]. In RN600/RA600, four genes in the five NRT families were up-regulated by *R. irregularis* inoculation, indicating that mycorrhizal *C. glauca* can promote nitrogen absorption to maintain ion balance and growth under NaCl stress. Overexpression of *PIP1-2* in apples could improve drought and salt tolerance, reduce MDA content, and increase antioxidant enzyme activity [66]. In our study, *R. irregularis* inoculation further increased the expression of TRINITY_DN11775_c0_g1 (*PIP1-2*) in *C. glauca* under NaCl stress. This shows that *PIP1-2* is important for mycorrhizal *C. glauca* to induce the NaCl tolerance.

### 4.3. Regulation of Transcription Factors by R. irregularis Inoculation under NaCl Stress

A transcription factor is a protein sequence that binds to specific DNA, which controls the transcription from DNA to mRNA, and is responsible for the expression of stress-activated genes for plant tolerance and adaptability [67]. bHLH, ERF, MYB, NAC, and WRKY are important transcription factor superfamilies in plants, which are involved in all aspects of plant growth and development, and have been proven to be important in salt tolerance [68]. We have found that a large number of genes related to cell wall pathways in plants are enriched during inoculation with *R. irregularis* under NaCl stress, which indicates that the development of cell walls and the degree of lignification are important to NaCl tolerance. Zhang et al. [69] found that inoculation with AMF could increase the salt tolerance by improving the development of roots’ cell walls and the degree of lignification of *Asparagus officinalis*. *MYB46* has been confirmed to be involved in the regulation of lignin synthesis in plants. The overexpression of *MYB46* in apples can improve plant tolerance to salt and drought [70]. Yogendra et al. [71] found that *MYB43* participated in the biosynthesis of plant secondary cell walls and promoted the synthesis and accumulation of phenylpropane metabolites. Our research also found that TRINITY_DN5601_c0_g2 (*MYB46*) and TRINITY_DN21562_c0_g2 (*NAC43*) were up-regulated in mycorrhizal *C. glauca* under NaCl stress, indicating that *R. irregularis* inoculation could promote the accumulation of lignin and the degree of lignification to improve NaCl tolerance of *C. glauca*. *WRKY1* and *WRKY19* are involved in hormone balance and salt adaptation in plants [72,73]. In our study, *R. irregularis* inoculation and NaCl stress jointly up-regulated TRINITY_DN6778_c0_g1 (*WRKY1*) and TRINITY_DN3531_c0_g1 (*WRKY19*), indicating that these two genes were important in the NaCl tolerance of mycorrhizal *C. glauca*.

## 5. Conclusions

The physiological and biochemical analysis showed that *R. irregularis* inoculation played an important role in promoting plant growth, regulating ion balance, and changing the activity of antioxidant enzymes. Transcriptome analysis of roots revealed that 1827 DEGs were affected by both *R. irregularis* inoculation and NaCl stress. Through the GO and KEGG enrichment analysis of these DEGs, it was found that *R. irregularis* inoculation improved the NaCl tolerance of *C. glauca* roots by regulating transcription factors, ion transport, antioxidase activity, carbohydrate metabolism, and cell wall. Our research focused on the ion transport, antioxidase activity, and transcription factors of *C. glauca* roots under NaCl stress, screening out the key genes related to these processes. The mycorrhizal *C. glauca* alleviated the damage of NaCl stress through *HAK5*, *KAT3*, *SKOR*, *PIP1-2*, *PER64*, *CPER*, *GLP10*, *MYB46*, *NAC43*, *WRKY1*, and *WRKY19*, providing molecular evidence that *R. irregularis* inoculation can assist *C. glauca* to relieve NaCl stress. This helps to visually express the salt tolerance mechanism induced by AMF and lays a foundation for the future use of AMF to enhance plant tolerance to salt stress.

## Figures and Tables

**Table 1:** Basic physiological and biochemical analysis of *C. glauca* ^1^.

	RN0	RA0		RN600		RA600
Fresh weight of shoot (g·plant^−1^)	4.65 ± 0.58 ^b^	16.23 ± 1.74 ^a^		0.59 ± 0.10 ^d^		2.79 ± 0.32 ^c^
Fresh weight of root (g·plant^−1^)	1.89 ± 0.38 ^b^	6.45 ± 1.00 ^a^		0.46 ± 0.08 ^c^		1.67 ± 0.20 ^b^
Height (cm)	26.9 ± 0.5 ^b^	51.3 ± 0.9 ^a^		11.0 ± 0.3 ^d^		23.8 ± 0.5 ^c^
Ground diameter (mm)	2.19 ± 0.39 ^b^	3.92 ± 0.59 ^a^		1.31 ± 0.28 ^c^		2.32 ± 0.46 ^b^
Colonization of arbuscule (%)	ND	39.30 ± 4.15 ^a^		ND		13.49 ± 6.56 ^b^
Colonization of hypha (%)	ND	92.26 ± 5.85 ^a^		ND		81.71 ± 6.58 ^a^
Colonization of vesicle & spore (%)	ND	33.77 ± 4.60 ^b^		ND		56.59 ± 9.67 ^a^
Concentration of Na^+^ (mg·g^−1^)	1.61 ± 0.39 ^d^	3.00 ± 0.48 ^c^		14.52 ± 0.92 ^a^		12.81 ± 0.71 ^b^
Content of Na^+^ (mg)	1.02 ± 0.16 ^b^	6.48 ± 1.81 ^a^		2.48 ± 0.26 ^b^		8.29 ± 1.22 ^a^
Concentration of Na^+^ in soil (g·kg^−1^)	0.13 ± 0.03 ^c^	0.04 ± 0.00 ^c^		0.71 ± 0.16 ^a^		0.44 ± 0.13 ^b^
Content of K^+^ (mg)	16.06 ± 1.98 ^b^	44.73 ± 5.24 ^a^		2.03 ± 0.24 ^d^		8.89 ± 1.49 ^c^
Content of Ca ^2+^ (mg)	16.49 ± 4.52 ^b^	64.88 ± 11.99 ^a^		5.55 ± 0.46 ^c^		15.71 ± 2.59 ^b^
Concentration of Cl^−^ (mg·g^−1^)	5.26 ± 0.91 ^b^	2.00 ± 0.36 ^c^		12.01 ± 0.51 ^a^		11.43 ± 1.29 ^a^
Content of Cl^−^ (mg)	3.36 ± 0.67 ^bc^	4.52 ± 1.29 ^b^		2.06 ± 0.25 ^c^		7.41 ± 1.51 ^a^
Concentration of Cl^−^ in soil (g·kg^−1^)	0.02 ± 0.00 ^b^	0.02 ± 0.00 ^b^		3.23 ± 0.55 ^a^		2.64 ± 0.65 ^a^
Na^+^/K^+^	0.08 ± 0.01 ^c^	0.12 ± 0.03 ^c^		1.23 ± 0.07 ^a^		0.93 ± 0.05 ^b^
Na^+^/Ca ^2+^	0.06 ± 0.02 ^d^	0.10 ± 0.02 ^c^		0.60 ± 0.03 ^a^		0.41 ± 0.02 ^b^
Concentration of MDA (mmol·g^−1^ FW)	44.76 ± 8.90 ^bc^	34.66 ± 4.12 ^c^		69.03 ± 6.05 ^a^		52.42 ± 1.88 ^b^
Activity of POD (μg·g^−1^ FW·min^−1^)	88.25 ± 3.07 ^b^	39.65 ± 7.50 ^b^		260.09 ± 61.26 ^a^		258.77 ± 21.68 ^a^
Activity of SOD (U·g^−1^ FW·min^−1^)	254.53 ± 91.47 ^c^	237.61 ± 33.78 ^c^		655.01 ± 218.34 ^b^		1066.13 ± 54.52 ^a^
	NaCl stress		inoculation of *R. irregularis*		their interaction	
Fresh weight of shoot (g·plant^−1^)	**		**		**	
Fresh weight of root (g·plant^−1^)	**		**		**	
Height (cm)	**		**		**	
Ground diameter (mm)	**		**		**	
Concentration of Na^+^ (mg·g^−1^)	ns		**		**	
Content of Na^+^ (mg)	**		*		ns	
Concentration of Na^+^ in soil (g·kg^−1^)	*		**		ns	
Content of K^+^ (mg)	**		**		**	
Content of Ca ^2+^ (mg)	**		**		**	
Concentration of Cl^−^ (mg·g^−1^)	**		**		**	
Content of Cl^−^ (mg)	**		ns		**	
Concentration of Cl^−^ in soil (g·kg^−1^)	ns		**		ns	
Na^+^/K^+^	**		**		**	
Na^+^/Ca ^2+^	**		**		**	
Concentration of MDA (mmol·g^−1^ FW)	**		**		ns	
Activity of POD (μg·g^−1^ FW·min^−1^)	ns		**		ns	
Activity of SOD (U·g^−1^ FW·min^−1^)	**		**		**	

^1^ R, roots; N, nonmycorrhizal; A, inoculated with *R. irregularis*; 0, no NaCl stress; 600, 600 mM NaCl stress. The data are the means ± standard error (*n* = 3). Different superscript letters indicate significant differences among the means by Tukey’s test (*p* < 0.05). Two-way ANOVA: “*” indicates *p* < 0.05, “**” indicates *p* < 0.01, and “ns” indicates no significant difference (*p* ≥ 0.05).

## Data Availability

The data that support the findings of this study are available in NCBI at https://www.ncbi.nlm.nih.gov/bioproject/, reference number is PRJNA690646.

## Supplementary Materials

The following are available online at https://www.mdpi.com/article/10.3390/microorganisms10010015/s1, Figure S1: The redox system of *C. glauca* under different treatments, Table S1: Primers used for quantitative real-time PCR (qRT–PCR), Table S2: Sequencing information of each group, Table S3: Transcript splicing information statistics, Table S4: Unigene annotation statistics results, Table S5: The exact number and names of 88 GO pathways in RA0/RA600 and RN0/RN600.
