# Supplementary material for: Transcriptome Analysis of Arbuscular Mycorrhizal Casuarina glauca in Damage Mitigation of Roots on NaCl Stress
Source: Microorganisms. 2021 Dec 23;10(1):15. doi: 10.3390/microorganisms10010015 (PMC8780529; doi:10.3390/microorganisms10010015)
Supplement: Supplementary file 1 [file microorganisms-10-00015-s001.zip › microorganisms-1488605-supplementary.pdf]

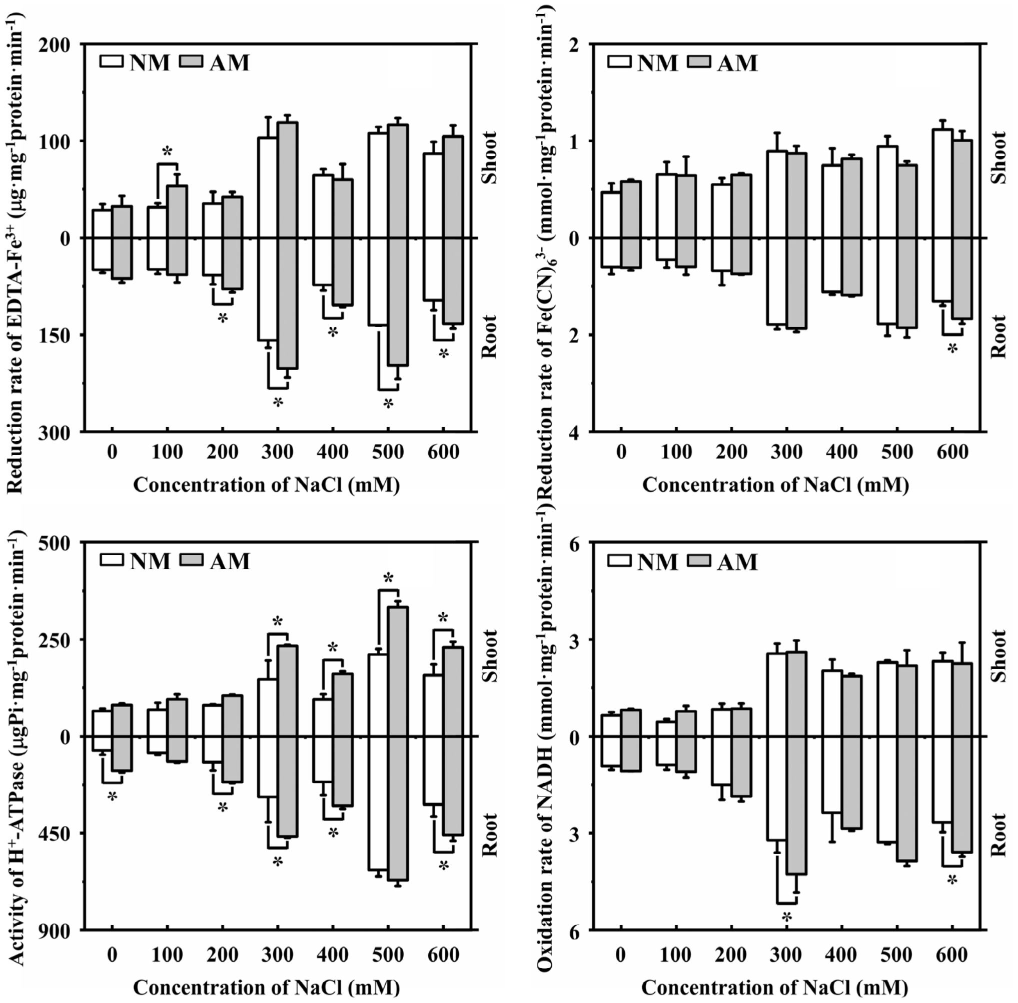

Figure S1. The redox system of *C. glauca* under different treatments. The redox system of plant plasma membrane is caused by the proton pump activity and the change of membrane potential. They not only effect the absorption of Na^+ and K^+, are also involved in plant stress resistance. Therefore, the salt concentration (600 mM) with the most significant difference between no AMF inoculum (NM) and inoculation of *R. irregularis* (AM) was screened out based on this standard, and the concentration was applied for subsequent experiments. The data are the means ± standard error (n = 3). "*" above the columns indicates significant difference among the means by Tukey's test ($p < 0.05$).

Table S1. Primers used for quantitative real-time PCR (qRT-PCR).

Gene Name	Forward Primer	Reverse Primer	Tm (℃)
CgHAK5	AAGAGGTAGTGGGTAGAGAGATTGA	GCACACCTTCACATTACGTTTTAGA	55.7
CgKAT3	TACTAGCATCTGACAACCCTTTTGA	CAGGACAAAATGAGGGAAAACAGAA	59.0
CgSKOR	GTCCATCAGCAAAGAAAATCTCCAA	ATTTGTCATGGAGAAGTGGATGAGA	55.0
CgNCL	TTACCTTGGTGATTGAGTTGAATGC	CGACGAAAATAAGCGGTTAAGAGAA	55.7
CgPER64	CAACAACCTTTGACAACACGTACTA	CAAATCTAAGCAAGGAAACGATGGA	55.7
CgCPER	CATTTGGAAAATTGTTGGATCTCGG	GTCGAAGTGATCTTTAACAGGCAAT	55.0
CgGLP10	AGTATAACATTCTCCGGGGTCAAAT	CAAACCTTGCTCCAAAGAAGAAGAA	55.0
CgTU20	GACTTGAAAACACCTTCAGGCTTTA	GAAGTTTGTTGCAGAGATCAAGGAA	63.3
CgEF1-α	CTTAACAAATCCAGCATCACC	CAGTCCTTGATTGCCACAC	59.0
CgTUB	TGTCAGTGGAGCAAACCCAA	CTATGACATTTGCTTCCGAAC	63.3

Table S2. Sequencing information of each group [1].

	Raw data	Clean data	N (%)	Q20 (%)	Q30 (%)
RN01	44861360	41544190	0.000349	97.67	93.79
RN02	41526138	38533420	0.000348	97.64	93.7
RN03	44316922	41022936	0.000345	97.83	94.1
RA01	45805556	42293288	0.000341	97.68	93.82
RA02	44137676	40548216	0.000346	97.52	93.42
RA03	43646560	40517392	0.000346	97.66	93.77
RN6001	46314674	43025000	0.000356	97.57	93.71
RN6002	44472614	41281282	0.000351	97.82	94.14
RN6003	48220388	44715732	0.000344	97.68	93.83
RA6001	48190362	44772044	0.000346	97.71	93.84
RA6002	43343100	40099568	0.00035	97.64	93.72
RA6003	46769586	43387978	0.00035	97.59	93.63
Average	45133745	41811754	0.000348	97.67	93.79

[1] R, roots; N, nonmycorrhizal; A, inoculated with *R. irregularis*; 0, no NaCl stress; 600, 600 mM NaCl stress; 1, 2, 3 represents triplicate of the same treatment. Raw data represents the total number of original reads in the sample, clean data represents the total number of high-quality reads in the sample, N (%) represents the percentage of fuzzy bases, and Q20 (%) represents the percentage of bases whose base recognition accuracy is over 99%, and Q30 (%) represents the percentage of bases whose base recognition accuracy is over 99.9%.

Table S3. Transcript splicing information statistics [1].

Contig	Transcript	Unigene
Total Length (bp)	410192391	84428372
Sequence Number	189861	58988
Max. Length (bp)	20358	20358
Mean Length (bp)	2160.49	1431.28
N50 (bp)	3329	2832
N50 Sequence No.	40873	9181
N90 (bp)	1063	525
N90 Sequence No.	120069	37569
GC%	41.14	40.77

[1] Transcript represents the sequence obtained after splicing; Unigene represents the sequence obtained after removing redundant sequences; total length (bp) represents the total length of the sequence; sequence number represents the total number of sequences; max. length (bp) represents the maximum length of the sequence; mean length (bp) means the average length of the sequence. Arrange all sequences from longest to shortest, then add the sequence lengths in this order. When the added length reaches 50% of the total length of the sequence, the length of the last sequence is N50 (bp). N50 Sequence No. means the total number of sequences with a length greater than N50. Arrange all sequences from longest to shortest, then add the sequence lengths in this order. When the added length reaches 90% of the total length of the sequence, the length of the last sequence is N90 (bp). N90 Sequence No. means the total number of sequences longer than N90; GC% means the GC content of the sequence.

Table S4. Unigene annotation statistics results [1].

Database	Number	Percentage (%)
NR	31457	53.33
GO	15702	26.62
KEGG	14075	23.86
Pfam	19496	33.05
eggNOG	30628	51.92
Swissprot	26164	44.35
In all database	6453	10.94

[1] Database represents the type of database, number represents the number of Unigenes successfully annotated in the database, percentage (%) represents the percentage of Unigenes that have successfully annotated in the database to the total Unigenes, in all databases represents the number of Unigene annotated in all databases.

Table S5. The exact number and names of 88 GO pathways in RA0/RA600 and RN0/RN600 [1].

Term	RA0/RA600	RN0/RN600
extracellular region	139	131
membrane	1467	1240
catalytic activity	2142	1802
carbohydrate metabolic process	265	241
intrinsic component of membrane	994	848
integral component of membrane	981	838
hydrolase activity, hydrolyzing O-glycosyl compounds	127	110
heme binding	123	112
hydrolase activity, acting on glycosyl bonds	138	123
tetrapyrrole binding	137	117
secondary metabolic process	67	52
polysaccharide metabolic process	88	80
transferase activity, transferring hexosyl groups	140	110
cell wall organization or biogenesis	88	86
oxidation-reduction process	442	391
oxidoreductase activity, acting on diphenols and related substances as donors, oxygen as acceptor	27	27
transferase activity, transferring glycosyl groups	174	146
drug catabolic process	74	63
glucosyltransferase activity	75	64
cellular polysaccharide metabolic process	59	56
hydrogen peroxide catabolic process	41	33
hydrogen peroxide metabolic process	41	33
iron ion binding	94	96
cell wall organization	63	60
antibiotic catabolic process	42	35
acid phosphatase activity	19	14
oxidoreductase activity, acting on diphenols and related substances as donors	29	28
cell wall	67	67
external encapsulating structure	67	67
oxidoreductase activity	499	440
oxidoreductase activity, acting on paired donors, with incorporation or reduction of molecular oxygen	101	100
UDP-glycosyltransferase activity	84	70
cofactor catabolic process	42	33
apoplast	38	39
external encapsulating structure organization	65	62
UDP-glucosyltransferase activity	66	55
cell wall macromolecule metabolic process	28	30
cellular carbohydrate metabolic process	77	77
protein kinase activity	352	292
cell periphery	243	228
glucan metabolic process	49	46
hydroquinone:oxygen oxidoreductase activity	18	19
cellular glucan metabolic process	48	45
phenylpropanoid catabolic process	18	19
lignin catabolic process	18	19
peroxidase activity	54	47
polysaccharide catabolic process	36	29

detoxification	64	53
monooxygenase activity	90	89
oxidoreductase activity, acting on peroxide as acceptor	55	47
cell wall polysaccharide metabolic process	21	23
cellular polysaccharide catabolic process	13	11
antibiotic metabolic process	43	38
secondary metabolite biosynthetic process	35	27
nutrient reservoir activity	16	15
plant-type cell wall organization	21	20
cellular response to toxic substance	54	45
lignin metabolic process	18	19
response to toxic substance	65	53
uronic acid metabolic process	39	31
glucuronate metabolic process	39	31
cellular glucuronidation	39	31
flavonoid glucuronidation	39	31
cellular detoxification	53	45
phenylpropanoid metabolic process	29	25
cellular oxidant detoxification	52	44
cofactor binding	260	220
reactive oxygen species metabolic process	44	36
glucan catabolic process	12	11
response to oxidative stress	59	51
xyloglucan metabolic process	11	11
plant-type cell wall organization or biogenesis	25	26
hemicellulose metabolic process	16	18
serine-type carboxypeptidase activity	19	16
plant-type cell wall	21	21
drug metabolic process	111	101
serine-type exopeptidase activity	20	17
xyloglucan: xyloglucosyl transferase activity	9	9
chitinase activity	15	14
copper ion binding	42	41
antioxidant activity	55	49
polysaccharide biosynthetic process	37	37
aminoglycan catabolic process	14	14
chitin metabolic process	14	14
chitin catabolic process	14	14
amino sugar catabolic process	14	14
glucosamine-containing compound metabolic process	14	14
glucosamine-containing compound catabolic process	14	14

[1] Supplementary material for Figure 2a.
